# Free-Ranging Macaque Mothers Exaggerate Tool-Using Behavior when Observed by Offspring

**DOI:** 10.1371/journal.pone.0004768

**Published:** 2009-03-10

**Authors:** Nobuo Masataka, Hiroki Koda, Nontakorn Urasopon, Kunio Watanabe

**Affiliations:** 1 Primate Research Institute, Kyoto University, Inuyama, Japan; 2 Department of Animal Science, Faculty of Agriculture, Ubon Rajathanee University, Bangkok, Thailand; Yale University, United States of America

## Abstract

The population-level use of tools has been reported in various animals. Nonetheless, how tool use might spread throughout a population is still an open question. In order to answer that, we observed the behavior of inserting human hair or human-hair-like material between their teeth as if they were using dental floss in a group of long-tailed macaques (*Macaca fascicularis*) in Thailand. The observation was undertaken by video-recording the tool-use of 7 adult females who were rearing 1-year-old infants, using the focal-animal-sampling method. When the data recorded were analyzed separately according to the presence/absence of the infant of the target animal in the target animal's proximity, the pattern of the tool-using action of long-tailed adult female macaques under our observation changed in the presence of the infant as compared with that in the absence of the infant so that the stream of tool-using action was punctuated by more pauses, repeated more often, and performed for a longer period during each bout in the presence of the infant. We interpret this as evidence for the possibility that they exaggerate their action in tool-using so as to facilitate the learning of the action by their own infants.

## Introduction

The population-level use of tools has been reported in various animals. One of the best known instances of this is the so-called “ant-fishing” by free-ranging chimpanzees (*Pan troglodytes*) [1]. Nonetheless, how tool use, including that of ant-fishing in chimpanzees, might spread throughout a population is still an open question [2]. There is some controversy as to whether the transfer of these cultural practices is accomplished across individuals by observational social learning or just by individual learning alone [3].

Although there is some disagreement about whether or not various forms of observational social learning play a role in the transmission, there is a general consensus among researchers that the recipient is solely responsible for the successful acquisition of the skill, and that the skill's donor does not have any active role in the transmission of cultural information. In the present paper, on the other hand, we present evidence which indicates the possibility that free-ranging adult long-tailed macaques (*Macaca fascicularis*) modify their action in tool-using so as to facilitate the learning of the action by their own infants. The behavior we observed was that of inserting human hair or human-hair-like material between their teeth as if they were using dental floss. We compared the pattern of the behavior in each of 7 adult females when her own infant was in her proximity and when any other group member was not in her proximity.

Our study of the tool-using behavior in a group of the macaques in Thailand started in 2004 and continues up to the present [4]. Whenever the material picked up by an animal is to be used as the tool, the animal subsequently grasps the hair taut between its two hands. Then, the animal inserts the taut hair between its open jaws, and the action ends when the animal closes its jaws to engage the taut hair, and pulls the hair sharply to one side by one hand and removes it from its mouth. Here a ‘bout’ of the tool-use is defined as starting at the moment of grasping the material with the hands and ending at the moment of completely removing it from the mouth. With this removing action, food, if present could be cleaned from between the teeth. Before removing the hair, the animal was often observed to repeatedly rapidly close and open (“snap”) its jaw to engage (clamp) the taut hair between its teeth. When this occurred, the number of times the animal clamped on the hair could be counted, calling it the number of snaps. Subsequent to the occurrence of such snapping, moreover, the animal was often observed to remove the taut hair which was kept grasped between the two hands, to briefly look at it at about eye level, and to reinsert it in its mouth as before. When this was observed, it was defined as an occurrence of “reinsertion” in a given bout. “Reinsertion” might be repeated in that bout: after reinserting the hair, the animal might repeat the same action and take out the hair again while grasping it with two hands. That bout continued until the animal finally pulled out the reinserted hair to one side using one hand. In each such bout, the number of occurrences of reinsertion as well as the number of occurrences of snapping while the hair was inserted could be counted. The length of each bout could also be measured by counting the number of frames of the video which were required to record from the onset until the end of the bout. In addition, the number of occurrences of “removing of the hair from the mouth” was computed in each bout as attempts to clean the teeth. It could be counted as ‘X+1 (X = 0, 1, 2,,,)’ in a given bout when the number of occurrences of reinsertion was ‘X’ in the bout.

When a bout ended, perhaps on the completion of the cleaning of the teeth, the animal abandoned the material onto the ground on some occasions. If this was observed, the tool-use ‘episode’ ended, during which a single bout of the activity was undertaken. Alternatively, however, the animal again grasped the material with the two hands and began another bout with an interval of no more than 1 second or so. Then, that episode continued until the animal finally abandoned the stimulus. Thus, the number of ‘bouts’ in the episode could be counted. Also, the number of frames of the video which were required to film from the onset of the first bout until the end of the final bout was defined as the total duration of that episode. If only a single bout was included in a given episode, the duration of the episode coincided with the duration of the bout. In addition, the total number of occurrences of “removing the hair from the mouth” in the episode was computed as an index of the frequency of cleaning attempts in the episode.

## Results

**Figure 1:**
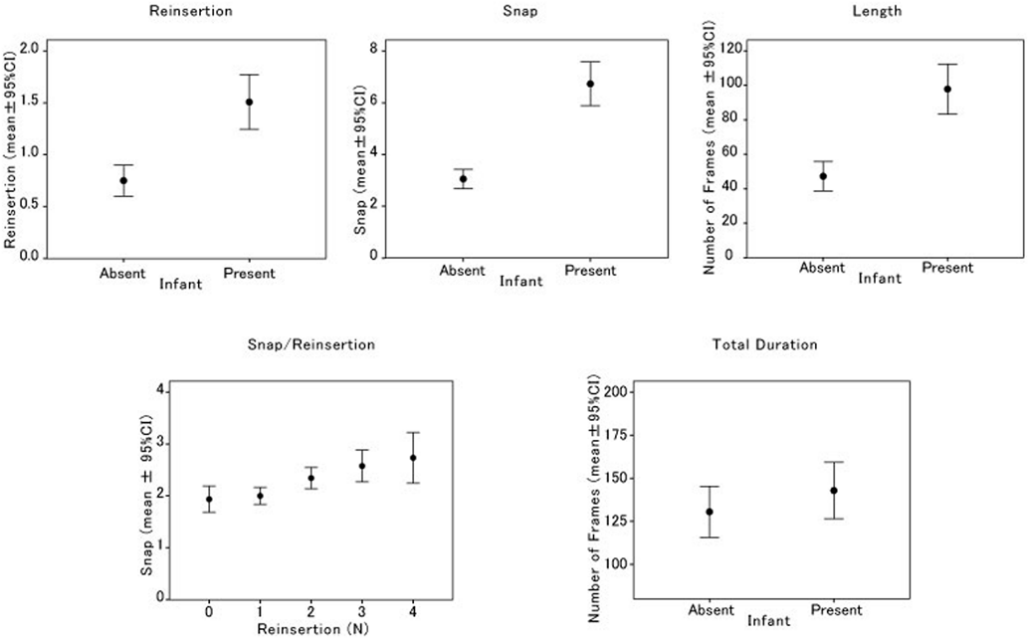
Summary of results of the analyses. Average scores of number of occurrences of reinsertion in a given tool-using bout (Reinsertion), of number of occurrences of snapping during each insertion (Snap), of length of each bout (Length), of overall mean number of snaps during each insertion as a function of number of occurrences of reinsertion in a tool-using bout (Snap/Reinsertion), and of total duration of a given tool-using episode (Total Duration) are computed across target adult females when the infant was in her proximity and when the infant was absent.

Results of the analyses are summarized in Figure 1. When the average number of occurrences of reinsertion in a given bout of the tool-use was computed across subjects, a likelihood-ratio test revealed that the score when the infant was in the proximity of the target mother was greater than that when the infant was absent (*χ* ^1^_2_ = 22.201, *p*<0.0001). Similarly, the average number of jaw snaps during each insertion of the stimulus was greater when the infant was present as compared to when the infant was absent (*χ* ^1^_2_ = 123.6, *p*<0.0001). The average duration of a given bout when the infant was present was longer that that when the infant was absent (*χ* ^1^_2_ = 44.51, *p*<0.0001). In a given bout, the number of occurrences of reinsertion was found to positively correlate with the number of jaw snaps during each insertion (Pearson's correlation = 0.232, *n* = 355, *p*<0.01). In a given bout, both the number of occurrences of reinsertion and the number of jaw snaps were found to positively correlate with the duration of the bout (Pearson's correlation = 0.770, *p*<0.001; 0.417, *p*<0.001; *n* = 355, respectively). The average duration of a given episode, on the other hand, did not differ significantly when the infant was present compared to when it was absent (*χ* ^1^_2_ = 1.592, *p* = 0.2071) because the average number of bouts in a given episode when the infant was absent was greater than that when the infant was present (*χ* ^1^_2_ = 8.9008, *p* = 0.00285). The average number of occurrences of removal of the hair from the teeth in a given episode did not differ when the infant was present compared to when it was absent (*χ* ^1^_2_ = 1.0519, *p* = 0.3051, mean±95%CI = 3.38±0.40 when the infant was present, and 3.76±0.50 when the infant was absent).

## Discussion

**Figure 2:**
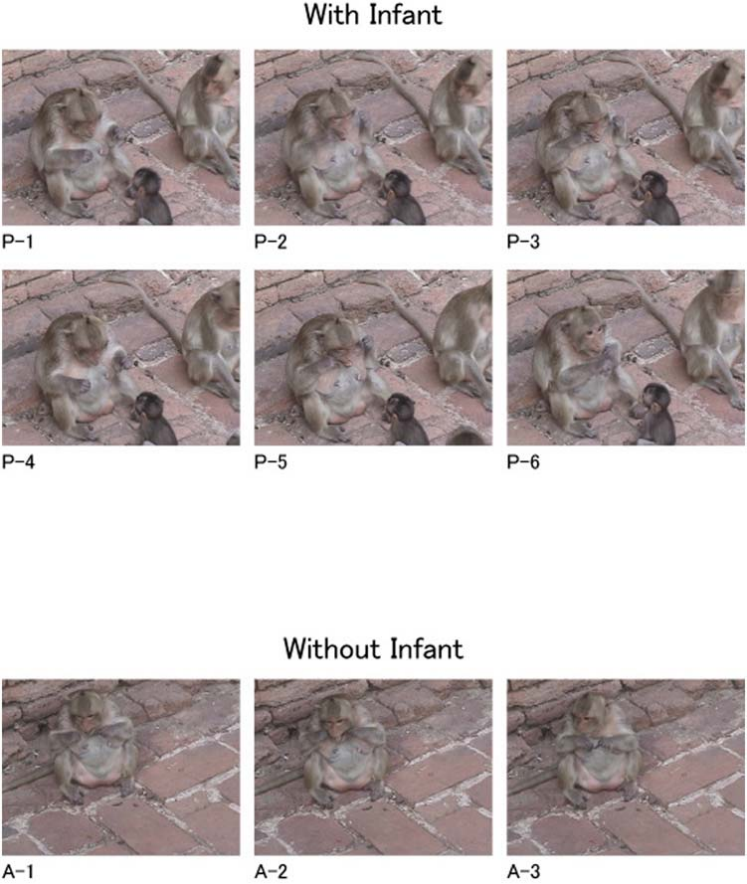
Typical sequences of the action of “flossing teeth”. (P 1 to 6) When her infant was in the proximity of an adult female (With Infant; P-1: Grasp the hair taut, P-2: Insert, P-3: Snap, P-4: Look at the hair, P-5: Reinsert, P-6: Pull out). (A 1 to 3) When no animal was in the proximity of an adult female (Without Infant; A-1 Grasp the hair taut, A-2: Insert, A-3: Pull out).

Overall, once the long-tailed macaque mothers (the target animals) started to use the stimulus as a tool, they devoted a similar amount of time to the stimulus regardless of whether or not their infant was present. However, as shown in Figure 2, the pattern of their action changed in the presence of the infants as compared with that in the absence of the infants so that the stream of tool-using action was punctuated by more pauses, repeated more often, and performed for a longer period during each bout in the presence of the infants.

As a possible factor affecting this difference, the activity of feeding by the animals per se is not considered likely because the present observations were undertaken at least 30 min after the end of the animal's final food-taking. Rather, it seems more likely that the behavioral difference is socially modulated, and influenced by the presence/absence of other animals in the proximity of the target animals. In this regard, the fact should be noted that only their infants were situated within arm's range of the target animals. Although no overt social interactions (occurrences of any facial expression or communicative movement) were observed in either the mothers or the infants, the influence of the presence of other group members than the infants did not appear to be a variable affecting this change.

As a possible explanation, one might assume that the mothers were more distracted when the infants were present and thus took longer to clean their teeth than when they were alone. However, the average duration of a given tool-using episode did not increase significantly when the infants were present. More importantly, the mothers' attempts to clean their teeth (as assessed by the number of times they removed the hair per episode) did not increase either when the infants were present. Actually, the average number of hair removal per episode when the infants were present was even smaller than that when the infants were absent. Rather, the change of the pattern of tool-using should be interpreted as a behavioral modification produced by the presence itself of the infants who were watching the mothers.

Concerning human mother-infant interactions, a series of experiments have revealed the fact that strikingly similar parental modifications in their actions, called motionese, can help infants to detect the meaningful structure of the actions [5], [6]. On the basis of observations of 51 and 42 mothers, respectively, who were demonstrating novel objects to their own infants whose ages ranged from 6 months to 13 months, it was found that the mothers tended to modify their infant-directed actions in various ways. They were likely to repeat the actions, to put longer pauses between actions and to exaggerate actions themselves. Such magnification of the movement or ‘looming’ has been argued so far to play an important role in educating the attention of human infants by attracting their attention due to the occlusion of other sensory information [7].

Indeed, such reasoning is confirmed by an analysis subsequently undertaken from an infant-like viewpoint by applying a model of saliency-based visual attention to such parental action [8], [9]. That analysis was conducted by scientists specializing in robotics originally for the purpose of investigating how such modifications contribute to the infant's understanding of the action. The results of their analysis showed that the model does not suppose any a priori knowledge about actions or objects used in the actions. Instead, it is able to detect and gaze at salient locations, which stand out from the surroundings because of the primitive visual features, in a scene. The model thus demonstrates which low-level aspects of parental actions are highlighted in their action sequences and could attract the attention of young infants, and also robots. Actually, a more recent experimental study [10] demonstrated infants' preference for motionese compared to adult-directed actions by presenting videos of both types of movement to 6- to 13-month-old infants. In the study, the participants showed evidence of such preferences even when demonstrators' faces were blurred in the videos.

Concerning macaques, unlike humans, there is no evidence for imitation under controlled conditions [3]. If we define imitation as the reproduction of the behavior of a model by an observer [11], most empirical studies have failed to show its occurrence in social groups. This could also be the case for the behavior of the monkeys in the present study. In order to explain the spread of the behavior in the group, therefore, we are forced to assume that animals may learn new behaviors from each other through simpler mechanisms than imitation. A typical instance of such reasoning is that its recipient's attention may be drawn to the environment or an object by the presence or interest of the donor itself, even in the absence of any form of intervention of social learning, for the transmission of cultural information. Under such circumstances, again, the modification of the action by the donor is as crucial as it is in the case of imitation because it profoundly affects the likelihood of the recipient acquiring a new behavior, which must be worked out by the recipient itself. The chance that the recipient's resulting behavior comes to resemble the donor's due to environmental or object constraints appears to be facilitated effectively by such modification of the behavior as we report here, which would eventually result in the population-level phenomenon of that behavior.

## Methods

The study group was inhabiting a small city, Lopburi, 154 km north of Bangkok, Thailand. In the center of the city stands the old Buddhist shrine of Prang Sam Yot in an open sandy area of approximately 50×50 m surrounded by three 20-m-wide roads and a railway. The present experiment was undertaken there. The area is included in the home range of the study group, which consisted of roughly 200 animals when the study was conducted in February, 2008. Because tourists often visit the shrine when it is open (between 9 a.m. and 5 p.m.), most of the group members were likely to stay there during this period. However, the study group does not spend night there, but in other woody areas at least 1 km away from the shrine. When the research started in 2004, we confirmed the tool-use in 9 adult female monkeys, who rode on the head of female tourists, pulled out their hair, and used it to “floss” their teeth [4]. Since then, the number of animals in which we have confirmed similar behavior has increased up to 50, all of which are adults.

During the study period, 7 females were rearing their approximately 1-year-old infants (3 males and 4 females). We chose all of these 7 females as target animals for the present study. The observation was undertaken by video-recording (30 frames per second) the tool-use of the adults in the area of the shrine. In order to control the variability of the material for the tool-use, we used hairs from a single type of human hairpiece. To provide the stimuli, on each day of observation, we scattered numerous hairs (approximately 20 cm long) that had been dissociated from the hairpieces around the study area early in the morning and waited for the target animals.

The data collection was undertaken using the focal-animal-sampling method. The collection starts with a focal animal, at least 30 min after than the final food-intake of that animal. When using the stimulus as a tool, the animal at first picks it up from the ground. Whenever such behavior is observed, our video-recording is started. When finishing the tool-use, on the other hand, the animal abandons the stimulus onto the ground, and we operationally defined this sequence of handling activity with the stimulus as the material for the teeth-flossing as an ‘episode’ of the tool-use.

In order to investigate whether the tool-using activity of a target animal was affected by the presence of other group members who were particularly naïve to the activity, we attempted to record the tool-using ‘episodes’ of the animal when her infant was present in her proximity and when no other animals were present in her proximity. The criterion was solely whether her infant alone remained present within arm's range as well as within the visual range of the target animal throughout a given episode, both animals being situated in a face-to-face position, or whether no animals remained present within such range throughout another given episode. In all, we were able to record 50 episodes where just her infant remained in the target animal's proximity and 50 episodes where no animals remained in the target animal's proximity. In addition, we recorded another 21 episodes during the study period. In these 21, however, animals other than the infant of the target animal entered into proximity with her during the tool-using activity (18 episodes), or the infant was not visually oriented toward the target animal (3 episodes). Thus, data concerning these cases were not included in further analyses.

The video-recording was performed using two video cameras. One of the two filmed the frontal view of the target animal. The tool-using behavior recorded by the videos was coded online by two highly trained coders independently from one another. They were not told the purpose of the present study. The detailed coding schema was essentially the same as that used in our previous study [12]. Overall interrater agreement was 97%. The other camera monitored the area proximal to of the animal. When the infant of the target animal was present in the proximity, the camera filmed its frontal view so that, by analyzing the videos recorded by this second camera and the camera monitoring the target animal, any occurrence of facial expressions and gestural movements could be recorded in both the infant and of the target animal. The occurrences were assessed again by the two raters. However, none of them reported any occurrence of such communicative behavior in the target animal or in the infant during any episode.

The research methodology complied with protocols approved by the guidelines (Guide for the Care and Use of Laboratory Primates, Second Edition) of Primate Research Institute, Kyoto University, Japan and the legal requirements of Thailand.
